# Irinotecan plus temozolomide in relapsed Ewing sarcoma: an integrated analysis of retrospective studies

**DOI:** 10.1186/s12885-022-09469-5

**Published:** 2022-03-31

**Authors:** Bi-Cheng Wang, Bo-Ya Xiao, Guo-He Lin

**Affiliations:** 1 Cancer Center, Union Hospital, Tongji Medical College, Huazhong University of Science and Technology, 1277 Jiefang Avenue, Wuhan, 430022 China; 2 Eastern Hepatobiliary Surgery Hospital, Second Military Medical University, Shanghai, 200438 China; 3 Department of Medical Psychology, Faculty of Psychology, Naval Medical University (Second Military Medical University), Shanghai, 200433 China; 4 Department of Oncology, the Second Affiliated Hospital of Anhui Medical University, Hefei, 230601 China

**Keywords:** Ewing sarcoma, Irinotecan, Temozolomide, Chemotherapy, Integrated analysis

## Abstract

**Background:**

The prognosis of patients with relapsed Ewing sarcoma is poor. In this study, we aimed to pooled-analyze the efficacy and safety of the combination of irinotecan and temozolomide in treating patients with relapsed Ewing sarcoma.

**Methods:**

PubMed, Cochrane CENTRAL, Web of Science, and EMBASE were systematically searched on September 27, 2021. The primary outcomes were rates of objective response and disease control, and the secondary outcomes were toxicities.

**Results:**

Six retrospective studies with 184 patients were enrolled in the analysis. The median age ranged from 14 to 21. The integrated rates were 44% (95% confidence interval [CI] 31–58) for objective response and 66% (55–77) for disease control. Grade 3–4 neutropenia, thrombocytopenia, and diarrhea occurred in 8% (3–16), 7% (3–11), and 8% (5–10) of chemotherapeutic cycles, respectively. 18% (7–32) and 6% (2–11) of patients suffered grade 3–4 neutropenia and thrombocytopenia after irinotecan plus temozolomide treatment.

**Conclusion:**

Irinotecan plus temozolomide combination chemotherapy showed antitumor activity and an acceptable safety profile in patients with relapsed Ewing sarcoma. More future prospective studies are needed to confirm the retrospective results.

## Highlights

1. Nearly 44% of relapsed Ewing sarcoma patients achieved an ORR after IT treatment.

2. Relapsed Ewing sarcoma treated with IT had an over 66% DCR.

3. IT chemotherapy caused low incidences of neutropenia, thrombocytopenia, and diarrhea in treating relapsed Ewing sarcoma.

## Introduction

Ewing sarcoma is a very rare tumor and usually occurs in childhood and young adults. Undifferentiated small round blue-cells are the main pathologic characteristics [1]. Histogenesis included immature reticulum, myogenous, endothelial, or undifferentiated mesenchymal cells [2]. Primary tumor treatment (surgery, radiation, or both) combined with chemotherapy significantly brings survival benefits [3–5]. The 5-year overall survival (OS) and event-free survival (EFS) could be 83 and 73% in localized Ewing sarcoma patients [5]. For patients with metastases, vincristine, doxorubicin/dactinomycin, and cyclophosphamide alternating with ifosfamide and etoposide chemotherapy are the preferred first-line drugs [6]. However, within two years, diseases in over two-thirds of patients progressed, and the 5-year OS and EFS were about 34 and 22% [4].

In order to increase the survival outcomes in advanced patients, a recently published prospective study had evaluated the activity and tolerability of irinotecan plus temozolomide as front-line chemotherapy in primary disseminated Ewing sarcoma. In the study, although grade 3–4 adverse events were observed in 3% of enrolled patients, the objective response rate (ORR) was 59%, with a 3-year OS of 36% and a 3-year EFS of 21% [7]. However, according to Asaftei’s report, first-line irinotecan and temozolomide combination chemotherapy failed to significantly prolong the survival outcomes in primary disseminated Ewing sarcoma patients.

In the second- or later-line setting for patients with recurrent and primary refractory Ewing sarcoma, therapeutic chemotherapies recommended by National Comprehensive Cancer Network (NCCN) guideline include cyclophosphamide + topotecan, irinotecan + temozolomide ± vincristine, cabozantinib, docetaxel + gemcitabine, and ifosfamide + carboplatin + etoposide [6]. Among these regimens, we commonly favor irinotecan plus temozolomide as a front choice for relapsed Ewing sarcoma in our institution (Wuhan Union Hospital).

The rEECur trial is the first randomized controlled study to compare the chemotherapeutic regimens in recurrent and primary refractory Ewing sarcoma. In this ongoing clinical trial, irinotecan plus temozolomide has a 20% response rate, a 4.7 months (95% CI: 3.4 to 5.7) progression-free survival (PFS), and a 13.9 months (95% CI: 10.6 to 18.1) OS, but the interim results find that the combination of irinotecan and temozolomide is less effective than topotecan plus cyclophosphamide, gemcitabine plus docetaxel, and high-dose ifosfamide [8].

However, after reviewing the published retrospective studies, we noticed that the effects of irinotecan plus temozolomide combination therapy were much better in relapsed Ewing sarcoma [9–14]. For instance, Palmerini et al. reported the data in 51 recurrent Ewing sarcoma patients. 13 patients received irinotecan plus temozolomide for first relapse/progression, while the combination chemotherapy was used at second or greater relapse/progression in the remainder. The overall ORR and disease control rate (DCR) were 34 and 71%, and the 1-year OS rate was 55%, independently of the line of chemotherapy [12].

Therefore, in this study, we synthesized the irinotecan and temozolomide chemotherapy data in treating patients with relapsed Ewing sarcoma in retrospective studies for comprehensively understanding the benefits and risks and future application.

## Materials and methods

We conducted this pooled analysis according to the Preferred Reporting Items for Systematic Reviews and Meta-analyses (PRISMA) guideline [15].

### Search strategy

A systematic search was performed in online databases (PubMed, Cochrane CENTRAL, Web of Science, and EMBASE) on September 27, 2021. The search terms included: (1) Ewing sarcoma, (2) irinotecan, and (3) temozolomide. References of relevant records in reviews were manually checked for more eligible studies.

### Selection criteria

All evaluable studies were assessed to meet the following inclusion and exclusion criteria.

Inclusion criteria:

1. Patients were diagnosed as relapsed Ewing sarcoma, including recurrent disease (defined as the patients who received up-front chemotherapy) and primary refractory disease (defined as the patients who progressed under upfront chemotherapy),
2. Patients were treated with irinotecan plus temozolomide chemotherapy,
3. Data of responses, survival outcomes, and/or toxicities were available.

Exclusion criteria:

1. Meeting abstracts
2. Case reports,
3. Basic or animal studies
4. Irinotecan and temozolomide concurrent with or followed by additional agents,
5. Data of Ewing sarcoma could not be separated from other types of tumors,
6. Non-English studies.

### Data extraction

ORR and DCR were the primary outcomes. ORR should be defined as the percentage of patients who achieved composite complete or partial responses, and DCR is defined as the overall rate of complete response, partial response, and stable disease. Treatment-related adverse events were the secondary outcomes. Bi-Cheng Wang and Guo-He Lin independently extracted detailed data from each article, including the name of the first author, year of publication, study design, number of patients, median age, doses, cycles, survival outcomes, responses, and toxicities. Any discrepancies were resolved by consensus.

### Risk of bias and statistical analysis

The risk of bias was evaluated by sensitivity analysis and Egger's test. Rates of objective response and disease control and incidences of toxicities were pooled-analyzed in a random-effects model owing to the single-arm data syntheses. All above analyses were conducted through R (version 4.1) software and the “meta” package [16, 17].

## Results

### Eligible studies and basic characteristics

**Fig. 1:**
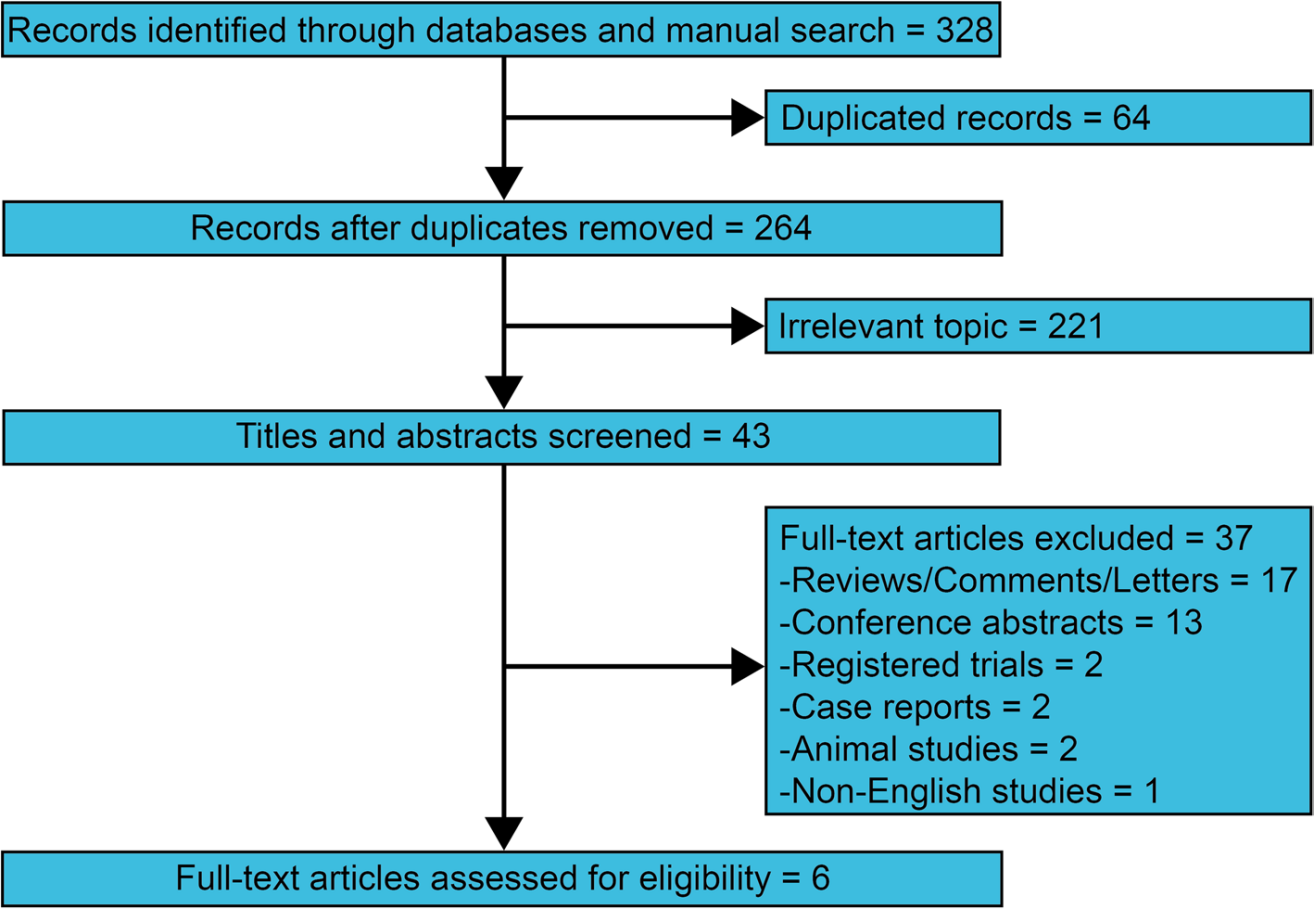
Flowchart of selecting the eligible studies

Through searching PubMed, Cochrane CENTRAL, Web of Science, and Embase, we identified 328 relevant records. 64 duplicated records were eliminated. 221 records were removed after screening the titles and abstracts. Furtherly, 37 full-text articles were eliminated because of reviews/comments/letters (*n* = 17), conference abstracts (*n* = 13), registered trials (*n* = 2), case reports (*n* = 2), animal studies (*n* = 2), and non-English studies (*n* = 1). Finally, six retrospective studies were enrolled in the pooled analysis (Fig. 1) [9–14].

Table 1 displayed the basic characteristics and details of the treatment schedules. The eligible studies were published from 2007 to 2021. A total of 184 relapsed Ewing sarcoma patients were collected. Two studies reported the data of both recurrent and primary refractory Ewing sarcoma [12, 14], while the other four showed the results of primary refractory Ewing sarcoma. All enrolled patients had been treated with up-front or adjuvant chemotherapy. The median age ranged from 14 to 21. The strategies of irinotecan included 10–20 mg/m^2^/day on day 1–5 and day 8–12, 10 mg/m^2^/day on days 1–5 (or expand to 10 days), and 40 mg/m^2^/day on day 1–5. While the therapeutic strategy of temozolomide was 100 mg/m^2^/day on days 1–5. The median number of cycles of the combination therapy ranged from 4 to 14.

**Table 1:** Basic characteristics and treatment schedules in eligible studies

First author	Year	Design	Number of patients	Median age (range)	Irinotecan	Temozolomide	Days of each cycle	Line of therapy	Median number of cycles (range)	Total cycles of therapy
Wagner	2007	Retrospective study	16	18 (7–33)	10–20 mg/m^2^/day on days 1–5 and days 8–12	100 mg/m^2^/day on days 1–5	every 21–28 days	> = 2	5 (1–17)	95
Anderson	2008	Retrospective study	25	15	10 mg/m^2^/day on days 1–5 (or expand to 10 days)	100 mg/m^2^/day on days 1–5	NA	NA	6	NA
Casey	2009	Retrospective study	20	19.5 (2–40)	10–20 mg/m^2^/day on days 1–5 and days 8–12	100 mg/m^2^/day on days 1–5	every 21 days	> = 2	7.5 (1–20)	154
Kurucu	2015	Retrospective study	20	14 (1–18)	20 mg/m^2^/day on days 1–5 and days 8–12	100 mg/m^2^/day on days 1–5	every 28 days	> = 2	14 (7–18)	97
Palmerini	2018	Retrospective study	51	21 (3–65)	40 mg/m^2^/day on days 1–5	100 mg/m^2^/day on days 1–5	every 21 days	> = 1	5 (1–31)	NA
Salah	2021	Retrospective study	52	20 (5–45)	40/50 mg/m^2^/day on days 1–5 or 20 mg/m^2^/day on days 1–5 and days 8–12	100 mg/m^2^/day on days 1–5	every 21 days	> = 2	4 (1–7)	236

*Abbreviations*: *NA* Data not available

### Responses

**Fig. 2:**
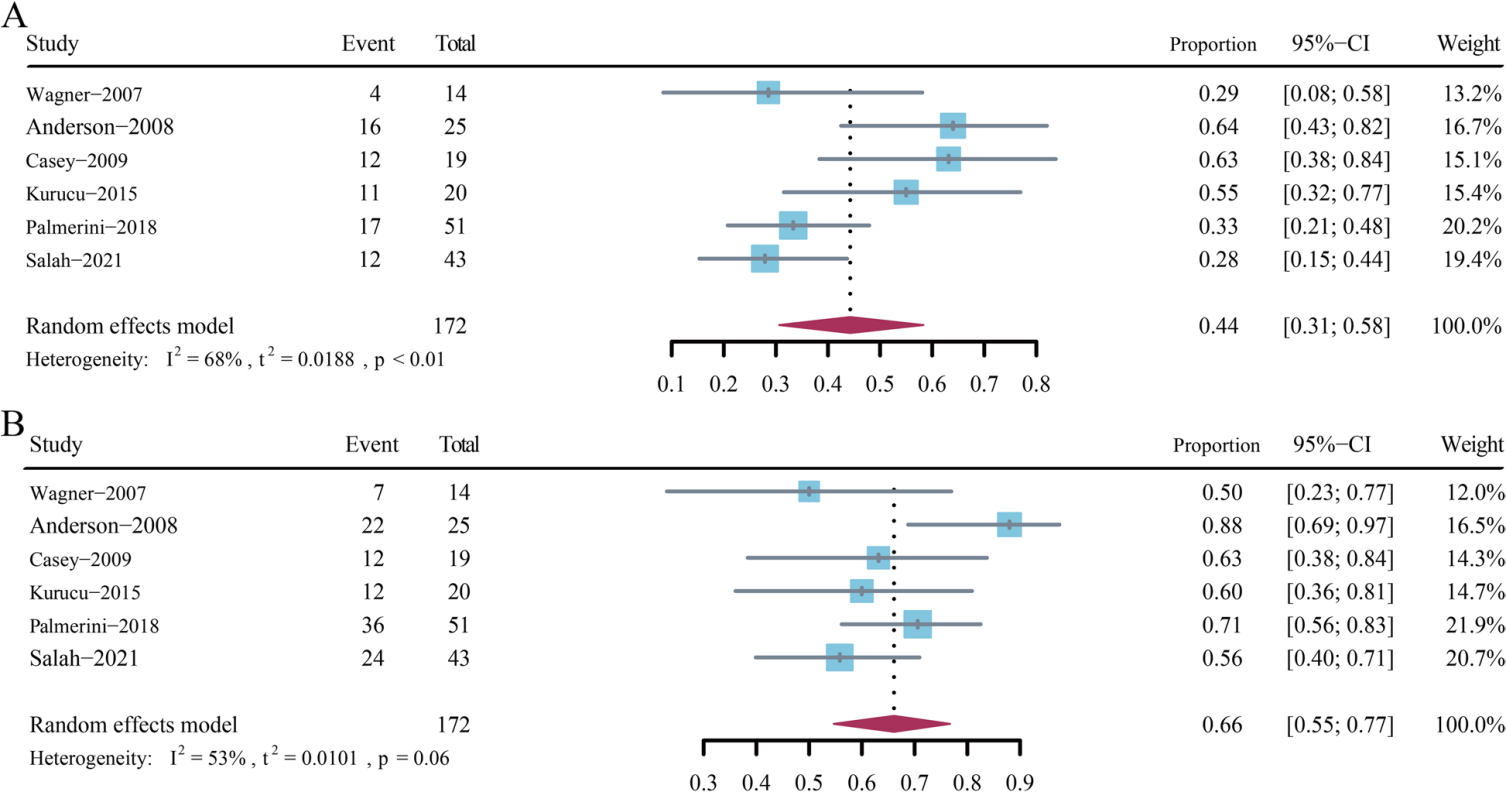
Pooled objective response rate (**A**) and disease control rate (**B**) in the analysis

Response data for 172 of the 184 included patients were available and were utilized to analyze ORR and DCR. The pooled rate of objective response was 44% (95% confidence interval [CI] 31–58%) (Fig. 2A), and the integrated DCR was 66% (95% CI 55–77%) (Fig. 2B).

### Survival outcomes

Table 2 showed the survival outcomes of irinotecan plus temozolomide in relapsed Ewing sarcoma. Median OS ranged from 12 to 14.1 months, and median PFS ranged from 3.8 to 8.3 months. 1-year OS and PFS rates were 55% and 44.4%, respectively. 6-month PFS rates ranged from 39 to 49%.

**Table 2:** Survival outcomes of irinotecan plus temozolomide in Ewing sarcoma

First author	Survival rates	Median OS (Months)	Median PFS (Months)
Wagner	NA	NA	4.7
Anderson	NA	NA	5.5
Casey	NA	NA	8.3
Kurucu	1-year OS: 54.2% 1-year PFS: 44.4%	12 (range 6–57)	5.5 (range 2–57)
Palmerini	1-year OS: 55% (95% CI 39–70) 6-month PFS: 49% (95% CI 35–63)	NA	3.9 (range 1–29)
Salah	6-month PFS: 39%	14.1	3.8

*Abbreviations*: *OS* Overall survival, *PFS* Progression-free survival, *NA* Data not available

## Toxicities

**Fig. 3:**
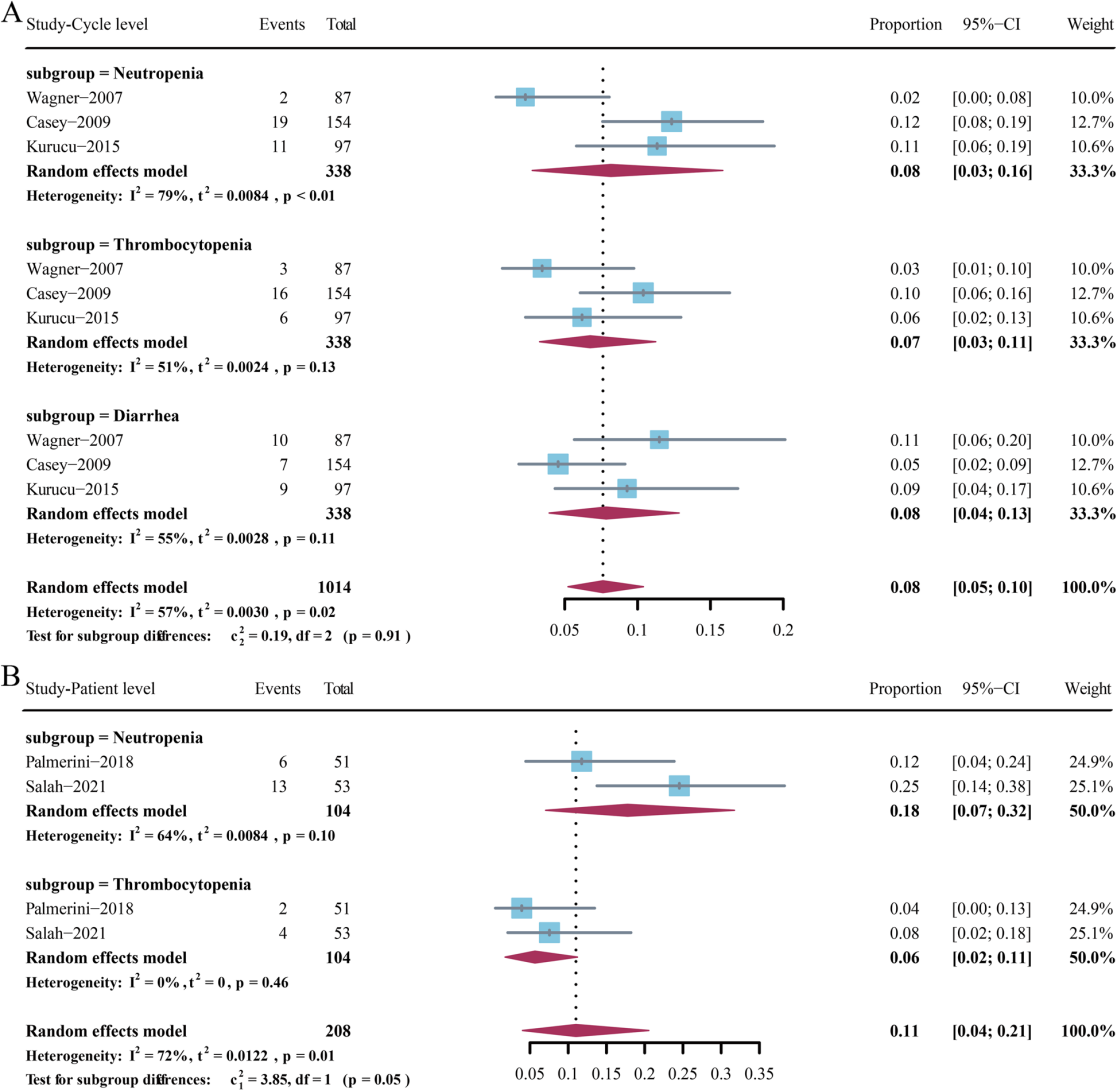
Pooled incidences of neutropenia, thrombocytopenia, and diarrhea in the cycle (**A**) and patient (**B**) levels

Treatment-related adverse events were extracted and integrated at the chemotherapy cycle and patient levels from all eligible studies (Fig. 3). In cycle level, data in three studies with 338 cycles were collected [9–11]. The pooled incidences of grade 3–4 neutropenia, thrombocytopenia, and diarrhea were 8% (95% CI 3–16%), 7% (95% CI 3–11%), and 8% (95% CI 5–10%), respectively.

In patient level, two studies with 104 patients were enrolled [12, 13]. The pooled incidences of neutropenia and thrombocytopenia were 18% (95% CI 7–32%) and 6% (95% CI 2–11%).

### Risk of bias

**Fig. 4:**
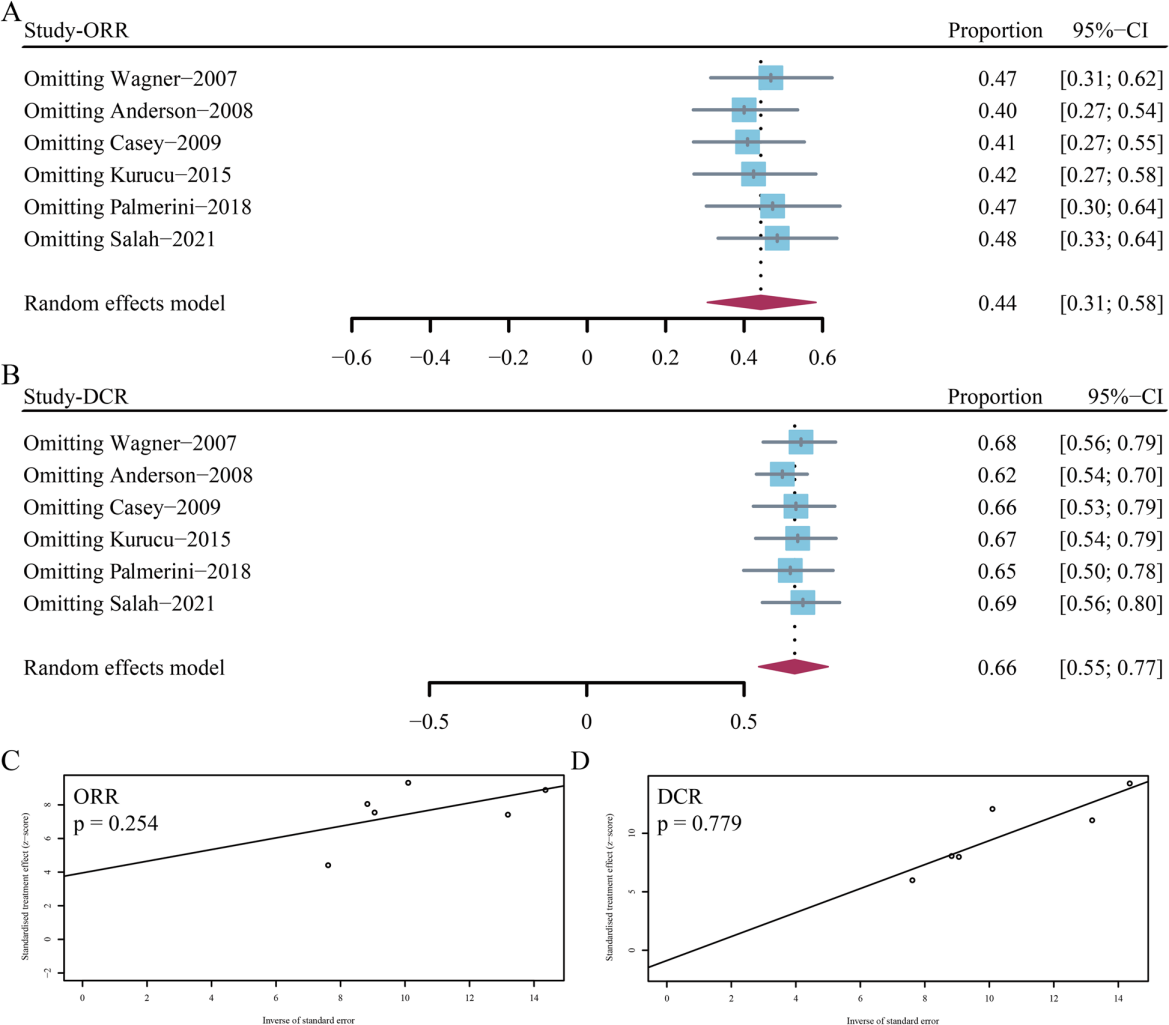
Sensitivity analysis and the risk of publication bias in the study. **A** and **C** Objective response rate; (**B** and **D**) Disease control rate

Figure 4A and B depicted the sensitivity analyses by omitting each enrolled study and showed highly consistent response rates. Egger’s tests did not find any publication bias among the studies (Fig. 4C and D).

## Discussion

In this analysis of retrospective studies, irinotecan plus temozolomide chemotherapy had an ORR of 44% and a DCR of 66% in treating relapsed Ewing sarcoma, with tolerable grade ≥ 3 treatment-related neutropenia, thrombocytopenia, and diarrhea.

In contrast, although 118 relapsed Ewing sarcoma patients received irinotecan plus temozolomide chemotherapy in the ongoing prospective study (rEECur trial), the ORR was only 20%, with a median PFS of 4.7 months and a median OS of 13.9 months [8]. Our pooled analysis of retrospective studies showed a much higher response rate (44%) versus the rEECur trial (20%). Regarding the toxicities, the most frequent treatment-related adverse event in the rEECur trial was diarrhea (17%), followed by vomiting/nausea (6%), fatigue (3%), and febrile neutropenia (3%) [8]. In our analysis, 18% and 6% of patients experienced grade 3–4 neutropenia and thrombocytopenia, and grade 3–4 diarrhea occurred in 8% of cycles. Owing to the incomplete data of the rEECur trial, the direct comparison of adverse events between prospective and retrospective studies is hard. We are eager to wait for the results in future prospective studies to show us more detailed information.

Besides the pooled results, the comparisons between irinotecan plus temozolomide and other second- or later-line chemotherapies deserve our attention.

### Irinotecan and temozolomide plus vincristine

In Raciborska’s study, 22 relapsed Ewing sarcoma patients received irinotecan (50 mg/m^2^/day on day 1–5) and temozolomide (125 mg/m^2^/day on day 1–5) plus vincristine (1.5 mg/m^2^/day on day 1). Median cycles were 4.1 per patient. Even the ORR was 54.5% and the DCR was 68.2%, the median time to disease progression was only three months (range 1.1 to 37.1) [18]. Compared with irinotecan plus temozolomide dual-drug chemotherapy, the triple-drug regimen showed a comparable effect and tolerability.

### Poly (adenosine diphosphate ribose) polymerase (PARP) inhibitors combined with irinotecan and/or temozolomide

Researchers have tried to combine PARP inhibitors (including niraparib and talazoparib) with irinotecan and/or temozolomide in Ewing sarcoma to elevate the response rates and survival outcomes [19, 20]. When patients were treated with niraparib with mono-drug chemotherapy, the median PFS was 9.0 weeks in the low-dose temozolomide group and 16.3 weeks in the irinotecan group [20]. In addition, talazoparib combined with irinotecan showed an ORR of 10.3% in solid tumors (Ewing sarcoma: 53%). The incidences of talazoparib plus irinotecan-related grade 3–4 neutropenia, thrombocytopenia, febrile neutropenia, and diarrhea were 78%, 42%, 24%, and 21%, respectively [19]. Even though PARP inhibitors combined with irinotecan or temozolomide were feasible and active in patients with Ewing sarcoma, detecting the combination of PARP inhibitors, irinotecan, and temozolomide is necessary. In Federico’s study, the authors displayed the benefits and risks of talazoparib plus irinotecan and temozolomide. The ORR was 25%, the most common grade 3–4 hematologic adverse events were neutropenia (31%) and thrombocytopenia (31%), and the most common grade 3–4 non-hematologic adverse events were febrile neutropenia (14%) and diarrhea (7%) [19]. Based on the published data, adding PARP inhibitors to irinotecan plus temozolomide chemotherapy was well tolerated but did not critically increase the response rates and survival outcomes in Ewing sarcoma patients.

### Cyclophosphamide plus topotecan

The study reported by Hunold detected the combination of cyclophosphamide (250 mg/m2/day on day 1–5) and topotecan (0.75 mg/m2/day on day 1–5) in 54 patients with relapsed Ewing sarcoma. With a median of 3 cycles of chemotherapy, the ORR and DCR were 32.6% and 59.1%, and the 1-year OS rate was 61% (95% CI 47–74%) [21]. Additionally, myelosuppression was reported in 76.9% of courses and only 4.4% of courses were associated with grade ≥ 3 infections [21]. In another retrospective study, 14 patients received first-line salvage therapy with cyclophosphamide 250 mg/m^2^ (on day 1–5) and topotecan 0.75 mg/m^2^ (on day 1–5). The median number of chemotherapeutic cycles was four (range 1–10). The results showed that Ewing sarcoma patients had a 23% (3/13 patients) ORR and a 77% DCR (7/13 patients) with manageable toxicities [22]. Accordingly, cyclophosphamide plus topotecan had a similar efficacy versus irinotecan plus temozolomide.

### High-dose ifosfamide

High-dose ifosfamide could be another treatment option for recurrent or advanced Ewing sarcoma patients. In Ferrari’s study, 37 Ewing sarcoma patients (including 33 patients with metastatic recurrent disease and four patients with progression during neoadjuvant chemotherapy) were administrated with 15 g/m^2^ ifosfamide. The median age was 17 years (6–45 years). 44% and 76% of patients achieved an ORR and a DCR, respectively. Nevertheless, extremely high incidences of grade 4 neutropenia (97% cycles) and thrombocytopenia (54% cycles) were observed [23].

### Cabozantinib

Cabozantinib is a MET and VEGFR2 inhibitor. Italiano et al. conducted a multicenter phase 2 clinical trial to investigate the activity of cabozantinib in advanced Ewing sarcoma [24]. 26% of 45 patients achieved an ORR. The median PFS was 4.4 months (95% CI 3.7–5.6 months) and OS was 10.2 months (95% CI 8.5–18.5 months). In 61 (68%) of 90 patients, at least one treatment-related severe adverse event was reported. However, there is a lack of direct comparison between cabozantinib and irinotecan plus temozolomide chemotherapy. It could be hard to deduce whether target therapy or combination chemotherapy is optimal.

### Docetaxel plus gemcitabine/Ifosfamide and carboplatin plus etoposide

The NCCN guideline has recommended docetaxel (75–100 mg/m^2^/day on day 8) + gemcitabine (675 mg/m^2^/day on days 1 and 8) and ifosfamide (1800 mg/m^2^/day on days 1–4) + carboplatin (400 mg/m^2^/day on day 1) + etoposide (100 mg/m^2^/day on days 1–4) chemotherapies as the second-line strategies [6]. In the studies cited by the NCCN guideline, docetaxel plus gemcitabine chemotherapy had a 29% ORR and ifosfamide plus carboplatin plus etoposide chemotherapy had a 51% ORR and an 84% DCR in relapsed sarcoma [25, 26]. In a prospective study reported by Mora, we noticed that Ewing sarcoma patients ≤ 18 years had a 74% (95% CI 56–97) of 5-year OS rate versus 31% for patients > 18 years [27]. It seemed that Ewing sarcoma inherently had better response rates in general in younger patients (because adult age and metastatic disease are poor prognostic factors for OS) when received docetaxel plus gemcitabine chemotherapy. Actually, more clinical trials are needed to confirm the efficacy of docetaxel plus gemcitabine/ifosfamide and carboplatin plus etoposide in treating relapsed Ewing sarcoma in the future.

### Limitations

Several limitations existed in this analysis. First, even this study showed the comparison between data in prospective clinical trials and our pooled analysis of retrospective studies, we could not demonstrate that the differences were statistically significant. Second, only retrospective studies were eligible. The enrolled articles mainly described retrospective assessments from single centers in which toxicity and response monitoring might not be necessarily standard. Moreover, retrospective studies might exert higher selection bias and lack strict evaluation. Third, the doses and administration schedules varied widely and more specific information. Fourth, outside of a clinical trial, it is easy to imagine that disease control could be overestimated if patients are not undergoing imaging at standard time points, and toxicity might be underappreciated if there are no routine toxicity assessments.

## Conclusion

Irinotecan combined with temozolomide is an effective and safe chemotherapeutic strategy for patients with relapsed Ewing sarcoma. Based on the results in this integrated analysis, we provided informative data for both clinicians and patients. In future prospective clinical trials, irinotecan plus temozolomide chemotherapy could be a preferred control strategy in searching for more effective therapies for Ewing sarcoma patients.

## Data Availability

All enrolled studies can be searched and downloaded from their official websites. 1. 10.1002/pbc.20697. 2. 10.1517/13543784.17.11.1703 3. 10.1002/pbc.22206. 4. 10.3109/08880018.2014.954070 5. 10.1080/0284186X.2018.1449250 6. 10.1007/s12094-020-02466-9.
